# Functional Assessment of Disease-Associated Regulatory Variants *In Vivo* Using a Versatile Dual Colour Transgenesis Strategy in Zebrafish

**DOI:** 10.1371/journal.pgen.1005193

**Published:** 2015-06-01

**Authors:** Shipra Bhatia, Christopher T. Gordon, Robert G. Foster, Lucie Melin, Véronique Abadie, Geneviève Baujat, Marie-Paule Vazquez, Jeanne Amiel, Stanislas Lyonnet, Veronica van Heyningen, Dirk A. Kleinjan

**Affiliations:** 1 MRC Human Genetics Unit, Institute of Genetics and Molecular Medicine, University of Edinburgh, Edinburgh, United Kingdom; 2 INSERM U781, Hôpital Necker-Enfants Malades and Université Paris Descartes-Sorbonne Paris Cité, Institute Imagine, Paris, France; 3 Service de Pédiatrie Générale, Université Paris Descartes, Hôpital Necker-Enfants Malades, Paris, France; 4 Departement de Génétique, Hôpital Necker-Enfants Malades, AP-HP, Paris France; 5 Service de Chirurgie Maxillo-Faciale et Plastique, CRMR des Malformations de la Face et de la Cavité Buccale, Hôpital Necker-Enfants Malades, Paris, France; EMBL Heidelberg, GERMANY

## Abstract

Disruption of gene regulation by sequence variation in non-coding regions of the genome is now recognised as a significant cause of human disease and disease susceptibility. Sequence variants in cis-regulatory elements (CREs), the primary determinants of spatio-temporal gene regulation, can alter transcription factor binding sites. While technological advances have led to easy identification of disease-associated CRE variants, robust methods for discerning functional CRE variants from background variation are lacking. Here we describe an efficient dual-colour reporter transgenesis approach in zebrafish, simultaneously allowing detailed *in vivo* comparison of spatio-temporal differences in regulatory activity between putative CRE variants and assessment of altered transcription factor binding potential of the variant. We validate the method on known disease-associated elements regulating *SHH*, *PAX6* and *IRF6* and subsequently characterise novel, ultra-long-range *SOX9* enhancers implicated in the craniofacial abnormality Pierre Robin Sequence. The method provides a highly cost-effective, fast and robust approach for simultaneously unravelling in a single assay whether, where and when in embryonic development a disease-associated CRE-variant is affecting its regulatory function.

## Author Summary

Cis-regulatory elements (CREs) play a vital role in gene regulation by providing spatial and temporal specificity to the expression of their target genes. Understanding how these regions of the genome work is of vital importance for human health as it has been demonstrated that genetic changes in these regions can result in incorrect gene expression, leading to a variety of human diseases. Functional characterization of putative CREs and the effects of mutations on their activity is currently a major bottleneck in many studies towards understanding the causes and mechanisms of disease and disease susceptibility. We describe a robust *in-vivo* approach using dual-colour reporter transgenesis in zebrafish for unambiguous assessment of the effects of disease-associated CRE mutations on CRE activity during the entire time-course of embryonic development. The highly efficient, cost-effective and modular design of the assay allows rapid analysis of several CRE-variants in parallel. We illustrate the robustness of our approach using examples of CRE-variants associated with a broad spectrum of genetic diseases including brain, limb, eye and jaw disorders. In a single assay the method can address where and when in development the CRE variant affects its activity, what potential target genes are misregulated by the change and what upstream trans-acting factors are likely to mediate this effect.

## Introduction

Cis-regulatory elements (CREs) such as enhancers are vital functional regions of the genome which determine where, when and at what levels their target genes will be expressed [1]. In keeping with their crucial role in the control of gene expression, CRE aberrations have been implicated as the cause of a variety of human diseases, genetic trait differences and predisposition to common diseases [2–3]. Modern approaches, such as next generation sequencing, now allow rapid determination of genetic variation between individuals to single nucleotide resolution. In contrast to the advancement offered by these high throughput methods for identification of sequence polymorphisms, progress towards an understanding of their precise role in disease etiology is hampered by a shortage of efficient methods for the functional characterization of observed variants. Identifying exactly which of the non-coding SNPs in a haplotype block have a regulatory function, and in what physiological context, has been complicated by some of the characteristic features of CREs: They can be located at very large distances (in some cases over a mega base) from their target genes; they can reside in gene deserts, or within the introns of- or even beyond- neighboring genes, making the prediction of their target genes difficult. Unlike coding mutations, the effect of non-coding sequence variation is difficult to predict. Moreover, CRE activities are often tissue- and stage-specific and highly dependent on the precise combination and stoichiometry of tissue-specific transcription factors [4]. The *in vivo* relevance, at the level of the whole organism, of approaches that utilize conventional assays in cell lines to test the effect of a disease-associated CRE variant is therefore highly questionable. The lack of efficient high-throughput and physiologically relevant assays for functional validation of tissue- and developmental-stage-specific CREs and identification of their target genes thus poses a real bottleneck in translating human non-coding variation into better understanding of disease mechanisms.

In our search for an efficient approach for characterizing regulatory variation in a vertebrate model system, we developed a versatile, streamlined assay pipeline using zebrafish (*Danio rerio*). Zebrafish embryos are transparent and develop rapidly and outside the mother, making it feasible to visualize specific tagged cell types in the living animal. They are well suited for transgenic manipulation since it is relatively easy to collect and inject large numbers of fertilized eggs [5]. These features make transgenic zebrafish a useful model for the characterisation of putative tissue-specific CREs in particular over the time-course of vertebrate development but also in adult tissues [6]. The use of multiple fluorescent reporters enables direct comparison between different cis-elements or different variants of the same element in the same cells of the same animal.

To determine the performance of the system, we tested a set of known disease-associated CREs in dual colour zebrafish reporter transgenics and demonstrate that the method is effective in revealing differences in functional output between variants in agreement with previously reported in mouse studies of these elements. We then applied the method to investigate a set of novel putative CREs with potential disease-associated variants in the genomic region of the *SOX9* gene. These variant elements, located at very long distances from *SOX9*, in the large gene desert between *SOX9* and *KCNJ2*, were identified based on their potential involvement in the congenital craniofacial malformation Pierre Robin Sequence (PRS) [7]. Consistent with their possible role in PRS aetiology, we show that these elements drive specific expression patterns in the craniofacial region of zebrafish and we reveal that the variant enhancer alleles found in the PRS patients have a variety of functional effects including: complete loss of activity; partial, tissue-specific loss of enhancer activity; and failure to maintain enhancer activity through development.

## Results

### Development of an efficient assay pipeline for rapid assessment of the functional relevance of sequence variants in potential cis-regulatory elements

Connecting a sequence variant in a non-coding genomic region to disease aetiology in the patient in whom the variant was detected, is currently a major bottleneck in human genetics.

**Fig 1:**
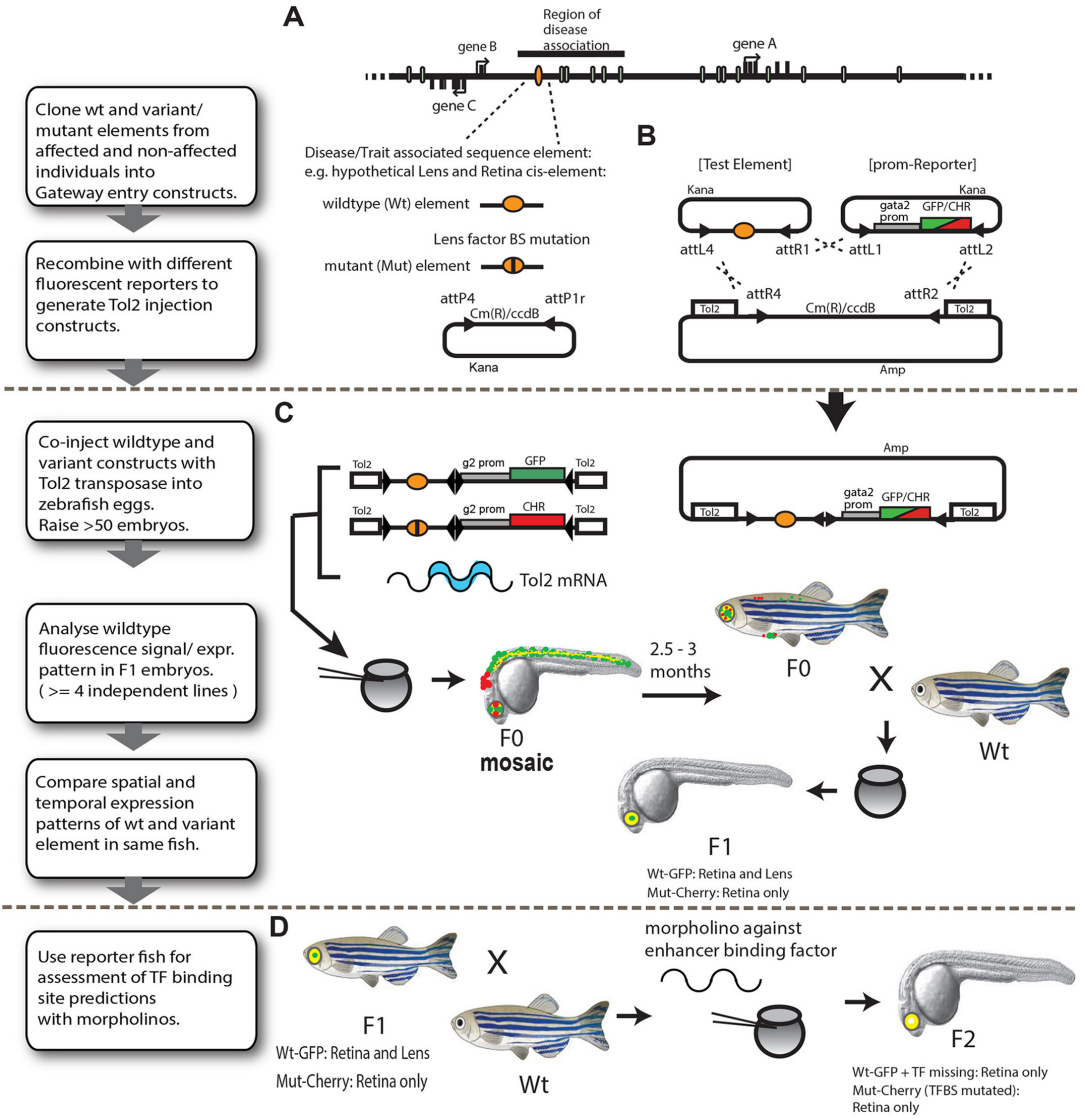
Schematic representation of the assay pipeline for *in vivo* characterisation of disease-associated cis-regulatory variants by dual-fluorescence reporter transgenic analysis in zebrafish. **(A-B)** The CRE bearing the disease-associated variant is amplified using genomic DNA, from the variant carrier individual. Both alleles of potential functional elements are cloned into a Gateway entry vector and recombined in a Multi-Gateway reaction with a reporter cassette of choice and a Tol2 inverted repeats containing construct to create the Cis-element-Reporter cassette flanked by Tol2 sites. **(C)** Co-injection of reporter vectors containing wild-type (Wt) and mutant (Mut) versions of the putative enhancer with Tol2 transposase-encoding RNA into early zebrafish embryos results in independent integration of the reporter cassettes. Transgenic founder fish (F0) are bred to establish transgenic lines. Expression patterns are examined in fish of F1 or later generations. Differences between wild-type and mutant elements can be compared directly in the same fish using the GFP and mCherry fluorescent reporters. **(D)** Disease-causing variants affecting a predicted transcription factor binding site can be validated by knockdown of the binding protein using morpholinos directed to the transacting factor.

To efficiently assess genomic regions harbouring putative disease associated sequence variants, we designed a versatile, robust and cost-effective assay pipeline which combines Gateway cloning technology and Tol2-mediated transgenesis to rapidly generate multiple dual-fluorescence reporter-transgenic zebrafish lines. The assay enables direct *in vivo* comparisons of the spatial and temporal activities of wild-type (Wt) and putative SNP or mutation (Mut) bearing CREs within the same animal by confocal imaging (Fig 1). The first step involves cloning of the genomic fragment(s) containing the putative cis-regulatory element using fast and highly-efficient Gateway cloning technology (Fig 1A and 1B). In cases where the disease involved is due to haplo-insufficiency of target gene expression, we were able to clone both wild-type and mutant fragments by PCR from heterozygous patient DNA. For fragments associated with quantitative traits, the phenotypic extremes of the trait will mostly segregate with homozygosity of the variant sequence. Nevertheless one can clone both variant fragments from an intermediate heterozygous individual or use a mix of the DNA of two homozygous extremes for single-step PCR cloning. Where patient DNA is not available the variant can be made via site-directed mutagenesis. The fragments of interest are inserted into an appropriate Gateway entry vector by inclusion of attB recombination sequences into the PCR cloning primers (Fig 1A and 1B). Sequencing is used to confirm the presence of each allele of the element of interest in the resulting clones and to exclude the presence of additional confounding mutations introduced during cloning. Both versions of the CRE are then recombined with different flavours of fluorescent reporter cassettes by a multi-gateway reaction into a destination vector containing Tol2 transposon sites for efficient zebrafish transgenic production (Fig 1B) [8]. The use of Gateway cloning vectors allows high-efficiency, rapid construction of enhancer-reporter constructs. This feature is especially advantageous for scaled-up testing of the functional relevance of a large number of putative CRE-variants identified in disease-association studies. Reporter cassettes encoding eGFP or mCherry driven from a gata2 minimal promoter were generally used, but can easily be exchanged for other promoter-reporter combinations. To exclude bias in detection levels between the eGFP and mCherry fluorophores we have conducted reporter cassette swaps for some of the elements, and found highly similar outcomes in all cases.

Both Tol2 constructs (bearing the Wt and Mut versions of the CRE) were co-injected with Tol2 mRNA into 1–2 cell stage zebrafish embryos (Fig 1C). Since many of the injected embryos (F0) show a variable extent of patchiness within their (mostly tissue-specific) expression patterns due to cellular mosaicism,the F0 founders are routinely grown to adulthood to generate stable lines. In our hands approximately 20 percent of injected embryos produce stable, dual reporter-expressing founder lines, thus yielding 7–10 independent founder lines in a single round of injection (of ~50 oocytes). The F0’s (we usually proceed with 3 to 5 independent F0’s per CRE) are then screened for reporter gene expression driven by the Wt and Mut versions of the CRE by out-crossing with non-transgenic fish of a standard laboratory strain. Tissue-specific CRE activities of the Wt and Mut alleles are scored in F1 embryos obtained from at least 3–5 independent F0 lines to eliminate any bias arising from the influence of site of integration of the transgene on the CRE activity. Tissues where CRE (Wt or Mut)-driven reporter gene expression is consistently observed in the progeny of >75% of independent F0 lines are scored as constituent parts of the activity domain of the CRE being analysed (S1 Table and S1 Fig). The expression pattern driven by the Wt and Mut versions of the CRE are visualised by confocal laser-scanning microscopy and compared unambiguously in the same animal through the entire time course of development of the F1 embryos (1–5dpf). Tissues and cell-types where the activities of the WT and Mut CREs completely overlap are observed as yellow in the merge channel, while the differences in CRE activities are revealed as green or red fluorescence. The assay thus provides complete and detailed spatial and temporal information about functional activity of the CRE and simultaneously reveals where and when in embryonic development this activity is affected by the presence of the disease-associated mutation. The combination of rapid cloning, high-efficiency, cost-effective transgenesis and dual-colour imaging makes the assay a very effective approach for rapid *in vivo* functional assessment of disease-implicated CRE-variants. Furthermore, in the case of novel CREs for which the target gene has not yet been established, comparisons can be made with the expression patterns of the zebrafish orthologs of potential candidates genes using RNA *in situ* hybridisation. It should however be kept in mind that subtle differences may exist between the expression of the zebrafish ortholog(s) and the human (or other species) gene the CRE is thought to regulate.

The most common established mechanism by which disease-associated sequence changes affect CRE function is by alteration or disruption of binding sites for tissue-specific transcription factors. The final part of our assay pipeline contains a convenient method for testing the validity of predicted disruptions of Transcription Factor Binding Sites (TFBS) by the disease-associated point-mutations (Fig 1D). F1 fish of selected reporter lines are bred to obtain dual-transgenic F2 embryos which are injected with morpholinos against the transcription factor whose binding is predicted to be affected by the mutation. The injected embryos are subsequently analysed for the effects of knock-down of the transcription factor on the expression pattern driven by the WT version of the CRE. The presence of the Mut CRE driving a different fluorophore within the same embryo allows unambiguous comparison of the effects produced by the transcription factor knock-down on the WT allele activity versus the effect of the altered binding site of the transcription factor as seen by the Mut-allele activity.

### Establishing robustness of the assay pipeline using known disease-associated enhancer variants

To test the robustness of our dual-fluorescence reporter transgenic assays we selected four disease-associated CREs from the literature for which point-mutations had been firmly established as the cause of human monogenic disease (Table 1).

**Table 1:** Known disease-associated CRE variants studied.

Element name (reference)	Target gene (HGNC no.)	Disease/trait (OMIM no.)	Expression pattern	Distance from promoter
SHH-SBE2 [9]	SHH (10848)	Holoprosencephaly (142945)	Forebrain	460 kb
ZRS [13]	SHH (10848)	Polydactyly (174500)	Limb buds	980 kb
IRF6-MCS-9.7 [15]	IRF6 (6121)	Orofacial clefting (608864)	Craniofacial	9.7 kb

**Fig 2:**
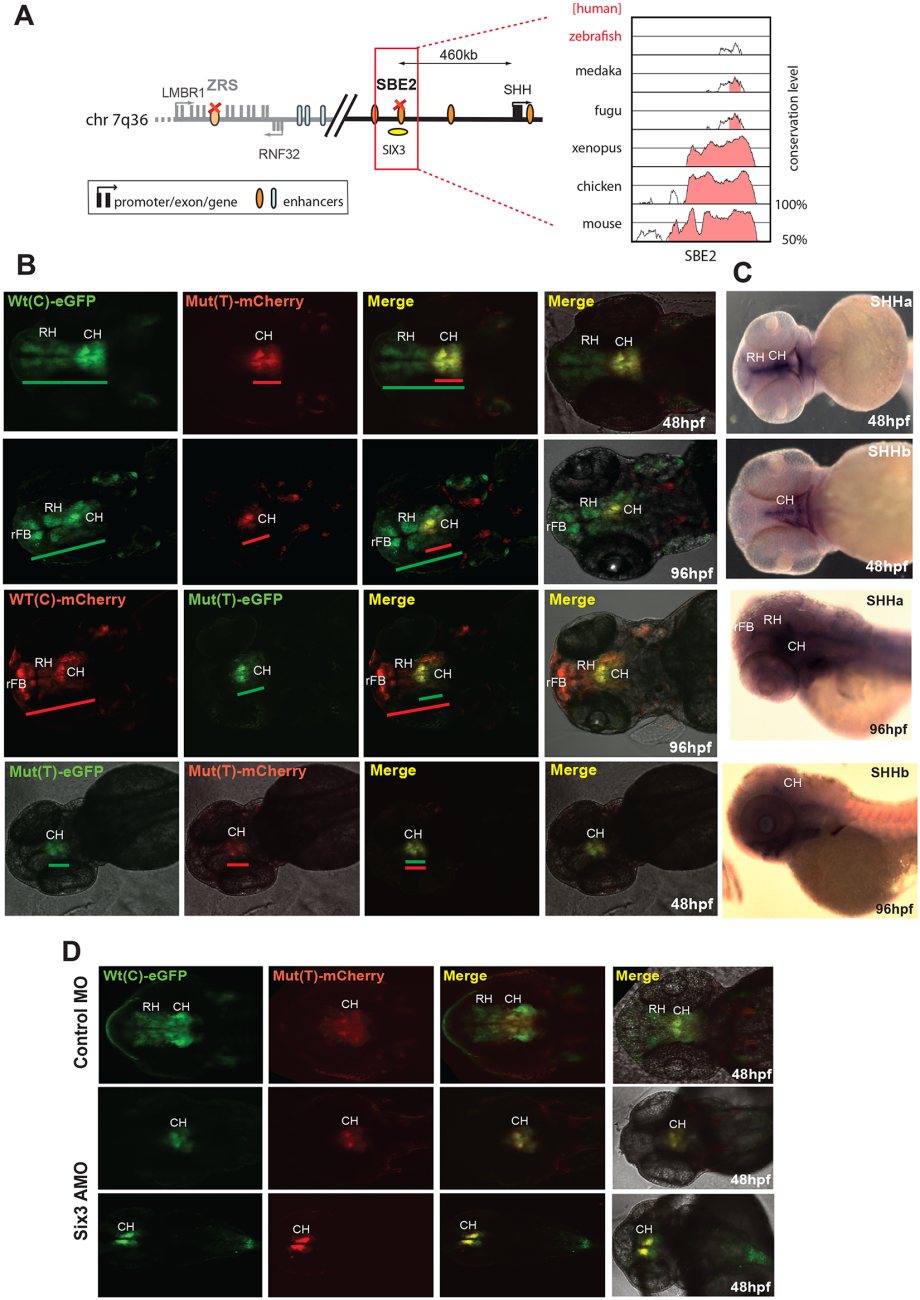
Dual-fluorescence transgenic analysis of a SHH enhancer SBE2 where a C>T change in a Six3 binding site has been identified in a patient with holoprosencephaly. **(A)** Regulatory landscape of SHH, depicting the location of the SBE2 enhancer. The sequence conservation plot on the right shows the low level of sequence conservation for SBE2 in the zebrafish genome. **(B)** SHH-SBE2 enhancer-driven F1 reporter transgenics. The wild-type allele Wt(C) drives expression in the rostral and caudal hypothalamus (long green or red bar) independent of the associated fluorophore (eGFP or mCherry). Expression from the mutant allele, Mut(T), is retained in the caudal hypothalamus (short green or red bar) but lost in the rostral hypothalamus (short green or red bar). Additional expression of the SBE2 Wt(C) allele in the rostral forebrain of later stage (96hpf) zebrafish embryos is also lost by the Mut(T) allele. **(C)** RNA *in situ* hybridisation analysis of *shha* and *shhb* expression at 48hpf and 96hpf of zebrafish embryonic development. The reporter gene expression pattern driven by Wt(C) allele significantly overlaps with the *shha* expression domain in the hypothalamus. **(D)** Morpholino knock-down of Six3 in SHH-SBE2 transgenic embryos mimics the effect of the Six3 binding site mutation in SBE2. F2 embryos bearing both SHH-SBE2 Wt(C) and Mut(T) alleles injected with either control morpholino (Control MO) or morpholino against both six3A and six3B (six3 AMO). Upon knockdown of six3, the hypothalamus expression driven by the Wt allele shrinks to overlap completely with the expression driven by the mutant allele (bearing a mutant six3 binding site). Wt: wild-type; Mut: mutant; hpf: hours post fertilization; MO: morpholino, RH: Rostral hypothalamus; CH: Caudal hypothalamus; rFB: Rostral forebrain.

The SBE2 element is a CRE that controls *SHH* (sonic hedgehog) expression in the developing forebrain (Fig 2A). A point mutation (C>T) in SBE2 was reported in a patient with holoprosencephaly, and in mouse transgenic experiments the mutation was shown to abrogate the activity of SBE2 in the rostral hypothalamus [9]. We tested the human Wt(C) and Mut(T) SBE2 alleles in our dual-colour zebrafish assay. Despite the low level of sequence conservation of human SBE2 in the zebrafish genome (Fig 2A), we observed activity of the Wt human SBE2 element in the developing rostral and caudal hypothalamus of transgenic fish. Simultaneous analysis of the holoprosencephaly-associated Mut allele in the same embryos unambiguously demonstrated the partial loss of activity in the rostral hypothalamus (Fig 2B). Dye-swap experiments between the Wt(C) and Mut(T) SBE2 alleles consistently showed loss of expression in rostral hypothalamus from the Mut(T) allele irrespective of the linked reporter fluorophore used, excluding any bias in detection levels between the GFP and mCherry fluorophores (Fig 2B). The Wt(C) allele also showed reporter signal in the rostral forebrain at 96hpf, which was lost when the Mut(T) allele was used (Fig 2B). This observation contrasts with the study of the mouse SBE2 element [9], where no activity of the enhancer was observed outside the hypothalamus. Some species-specific differences in CRE activity can thus be observed while employing these CRE-reporter assays. The reporter gene expression pattern driven by the Wt(C) SBE2 allele in the rostral and caudal hypothalamus significantly overlapped with the endogenous expression pattern of its target gene *shha* in the developing zebrafish embryo (Fig 2C). The C>T change in the SBE2 CRE had been demonstrated to occur in a SIX3 TFBS and disrupts SIX3 binding [9–10]. We knocked down Six3 by injecting morpholinos against Six3a and Six3b [11] into F2 transgenic embryos and the extent of Six3 depletion was assessed by immunoblot (S2 Fig). We observed that reporter expression driven by the Shh-SBE2 CRE Wt(C) allele shrunk to a more restricted domain in the caudal hypothalamus to match with the expression driven by the Mut(T) allele (Fig 2D). The expression domain driven by the Mut(T) allele, bearing a disrupted Six3 binding site, was not altered upon Six3 knockdown. Expression driven by both the alleles was unaffected upon injections of control morpholinos (Fig 2D). Our assay therefore demonstrates or confirms that the C>T mutation compromised Shh-SBE2 function by abrogating Six3 binding.

**Fig 3:**
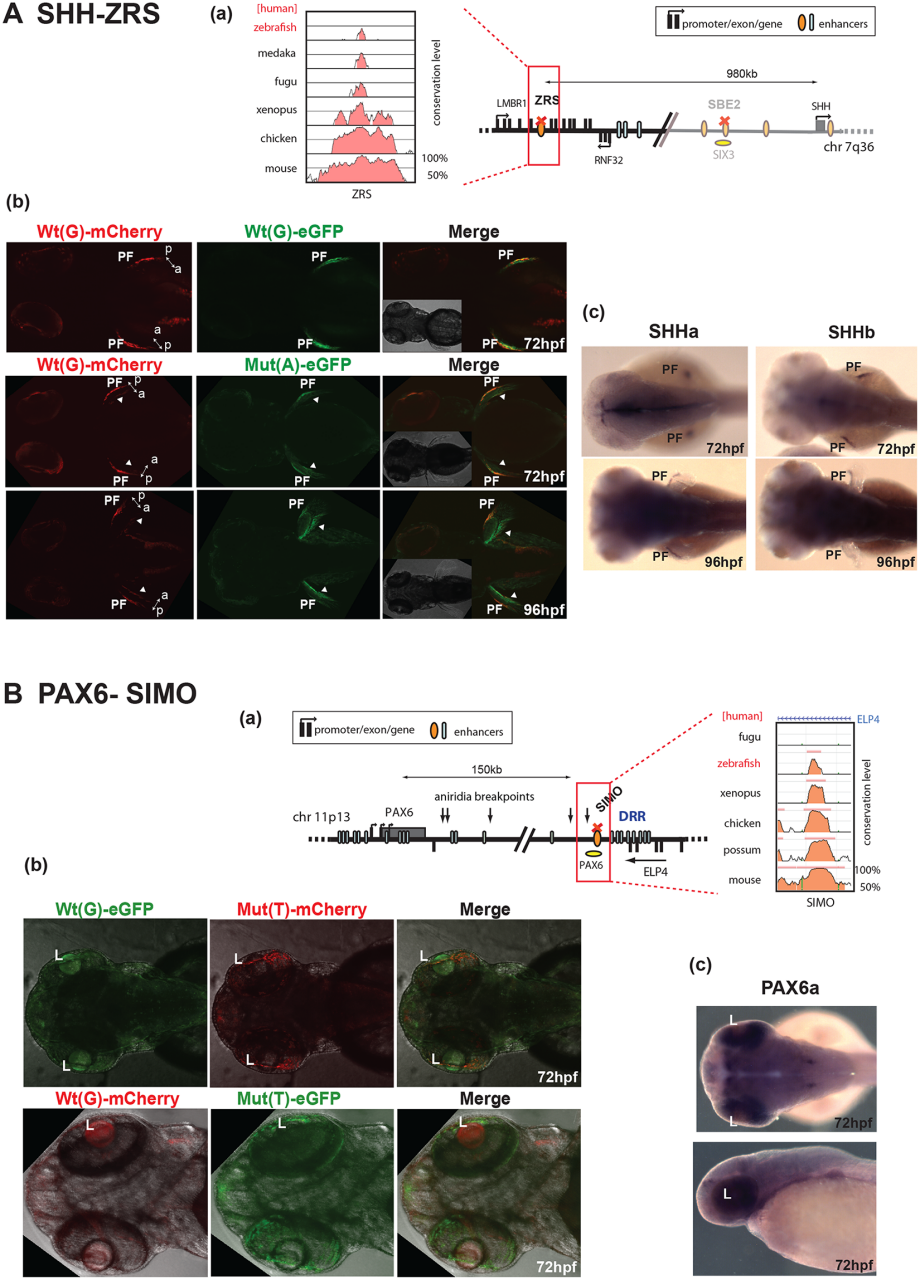
Dual-fluorescence transgenic analysis of established CREs with disease-associated point mutations. **(A)** SHH-ZRS. (a) The regulatory landscape of SHH, depicting the location of ZRS enhancer. The conservation plot on the left shows the low level of sequence conservation for ZRS in the zebrafish genome. (b) SHH-ZRS enhancer-driven reporter expression is shown at 72 hpf and 96 hpf. The top row shows the Wt(G) allele driving both mCherry and eGFP expression to equal measure in a restricted region of the developing pectoral fin (PF), coinciding with the expression domain of both *shha* and *shhb* in the developing pectoral fin (c). The Mut(A) allele drives expression at an additional site on the opposite edge of the fin. The ectopic expression (arrowheads) is increased by 96 hpf. The double-headed arrow indicates the anterior (a)—posterior (p) axial plane of the developing pectoral fin. **(B)** PAX6-SIMO. **(a)** The regulatory landscape of PAX6, depicting the location of SIMO enhancer. The conservation plot on the right shows the presence of sequence conservation for SIMO in the zebrafish genome. (b) PAX6-SIMO enhancer-driven reporter expression is shown at 72 hpf. In contrast to the Wt(G) element, and irrespective of the fluorophore used, the Mut(T) allele consistently fails to drive reporter gene expression in the developing lens (L). (c) RNA *in situ* hybridisation analysis of zebrafish *pax6a* at 72hpf showing the endogenous *pax6a* expression pattern in the developing eye with strong retinal expression and weaker lens expression overlapping with the reporter domain driven by the Wt(G). Wt: wild-type; Mut: mutant; hpf: hours post fertilization.

*SHH* encodes a powerful morphogen whose expression at different times and places during development is controlled by multiple CREs distributed over a 1Mb regulatory domain. The most distant of these—the ZRS (ZPA regulatory sequence)—controls expression that is restricted to the posterior mesenchyme of the distal limb bud (Fig 3A). A number of different point-mutations in the ZRS have been reported in patients with pre-axial polydactyly [12]. Among these, the Cuban mutation, a G>A change, has been shown to cause strong ectopic reporter expression in the anterior part of the limb-bud in transgenic mice [13]. Therefore this mutation appears to represent a gain of function in a CRE, rather than a loss of function as demonstrated for the SBE2. To test whether our assay can detect this altered ZRS function, we assayed the activity of the Wt(G) and Mut (A) human ZRS alleles. The Wt(G) allele drove expression of both eGFP and mCherry reporters in the zone of polarising activity (ZPA) of the developing pectoral fins (S3 Fig), at the same site where zebrafish both *shha* and *shhb* expression is normally seen (Fig 3Ac). This is consistent with the expression pattern driven by this CRE in mouse experiments [13]. We observed a mutation-driven expansion of reporter expression at 72 hpf, comparable to the changes reported in the mouse forelimb bud. By 96 hpf the additional domain of mutant ZRS-eGFP expression has become stronger and more obvious, strongly lighting up the anterior edge of the growing pectoral fin (Fig 3Ab). Thus our assay is able to detect a gain of function in CRE activity.

We next analysed a CRE from another developmental gene locus. The SIMO element regulates the expression of *PAX6* in the eye, and we have previously demonstrated that a point mutation in this CRE can cause the congenital eye malformation aniridia by disrupting an autoregulatory PAX6 binding site in the element [14]. Using dye-swap experiments we demonstrated that, in contrast to the Wt(G) element, and irrespective of the fluorophore used, the Mut(T) allele consistently fails to drive reporter gene expression in the developing lens (Fig 3Bb). This observation was in complete agreement with the results obtained when the Wt and Mut elements were tested using mouse transgenic experiments [14]. The reporter gene expression driven by the Wt(G) allele also overlapped with the expression pattern of the zebrafish homolog of its target gene (pax6a) (Fig 3Bc).

**Fig 4:**
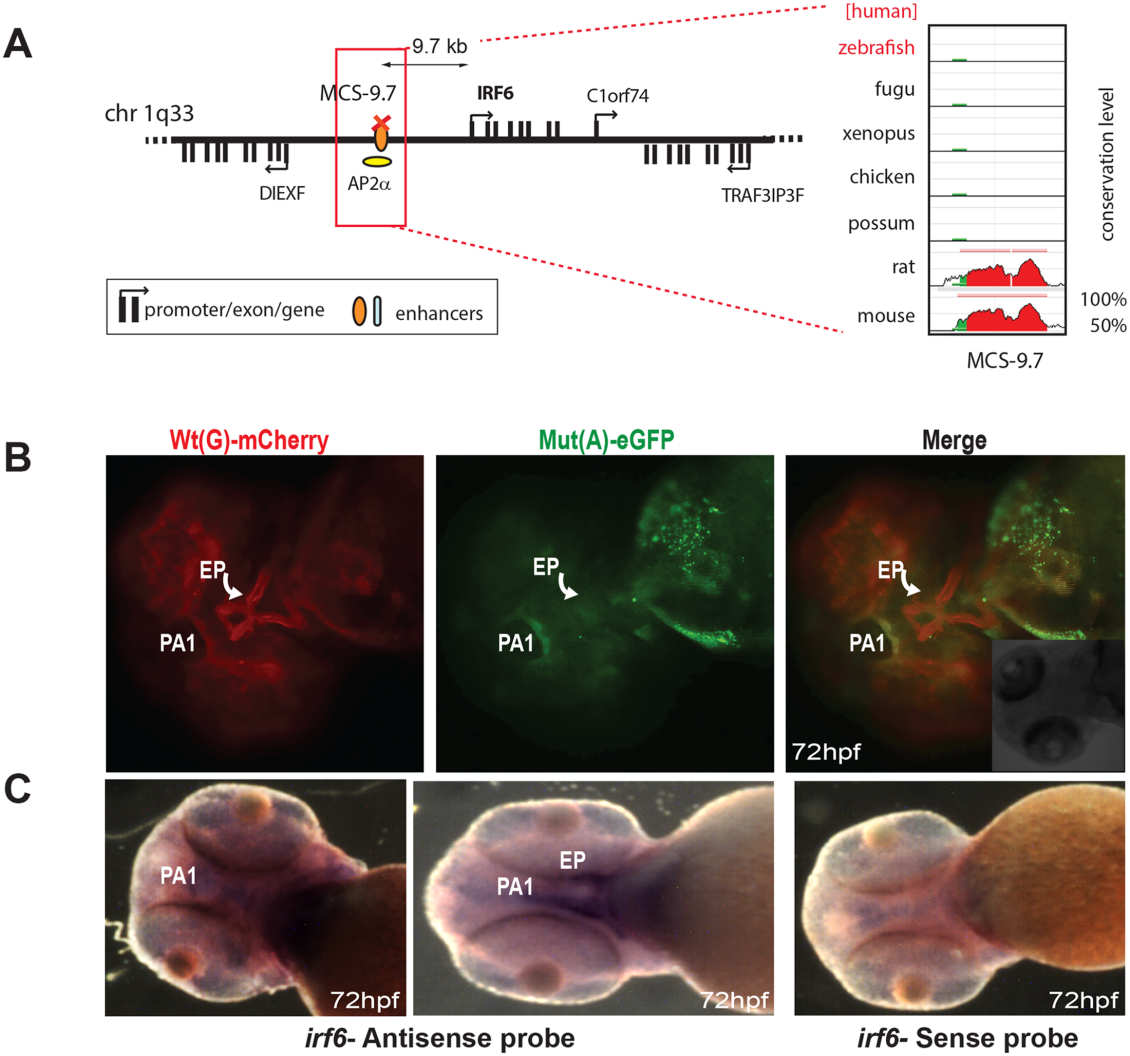
Dual-fluorescence transgenic analysis of IRF6 enhancer MCS-9.7 where a SNP (rs642961, G>A) in a TFAP2A (AP2α) binding site has been associated with cleft lip. **(A)** Regulatory landscape of IRF6, depicting the location of MCS-9.7 enhancer. The conservation plot on the right shows the absence of sequence conservation for MCS-9.7 in the zebrafish genome. **(B)** IRF6-MCS-9.7 enhancer-driven F1 reporter transgenics. The Wt(G) allele drives expression in the first pharyngeal arch (PA1) (arrow) and in the developing ethmoid plate (EP) (curved arrow). Mut(A) has lost EP expression but maintains PA1 expression. **(C)** RNA *in situ* hybridisation analysis of zebrafish *irf6* at 72hpf showing overlap of the reporter gene expression domain driven by Wt(G) allele with the endogenous *irf6* expression pattern in the developing jaw. Wt: wild-type; Mut: mutant; hpf: hours post fertilization.

The final known disease-associated non-coding variant we studied was a well-established polymorphic change associated with cleft lip, in an upstream regulatory element controlling the expression of Interferon Regulatory Factor 6 (IRF6) [15]. The minor allele mutant site (designated as SNP rs642961) has been shown to disrupt a TFAP2A binding site, but no comparative analysis had been carried out previously to show how the wild-type and variant enhancer functions differ [15]. The Wt(G) allele of this element drives expression in the first pharyngeal arch and in the ethmoid plate of transgenic fish, both sites where IRF6 is thought to play a key role during oral and palate development [16]. We found that expression driven by the Mut(A) transgene was maintained in the pharyngeal arch, but expression in the ethmoid plate was abolished (Fig 4B). Endogenous expression of zebrafish *irf6* was also detected in both the first pharyngeal arc and the ethmoid plate (Fig 4C). Our assay is thus able to demonstrate unambiguously the *in vivo* effect of a common disease-associated variant on enhancer activity.

### Functional characterisation of novel long-range CREs from the SOX9 locus carrying PRS-associated private sequence variants

**Fig 5:**
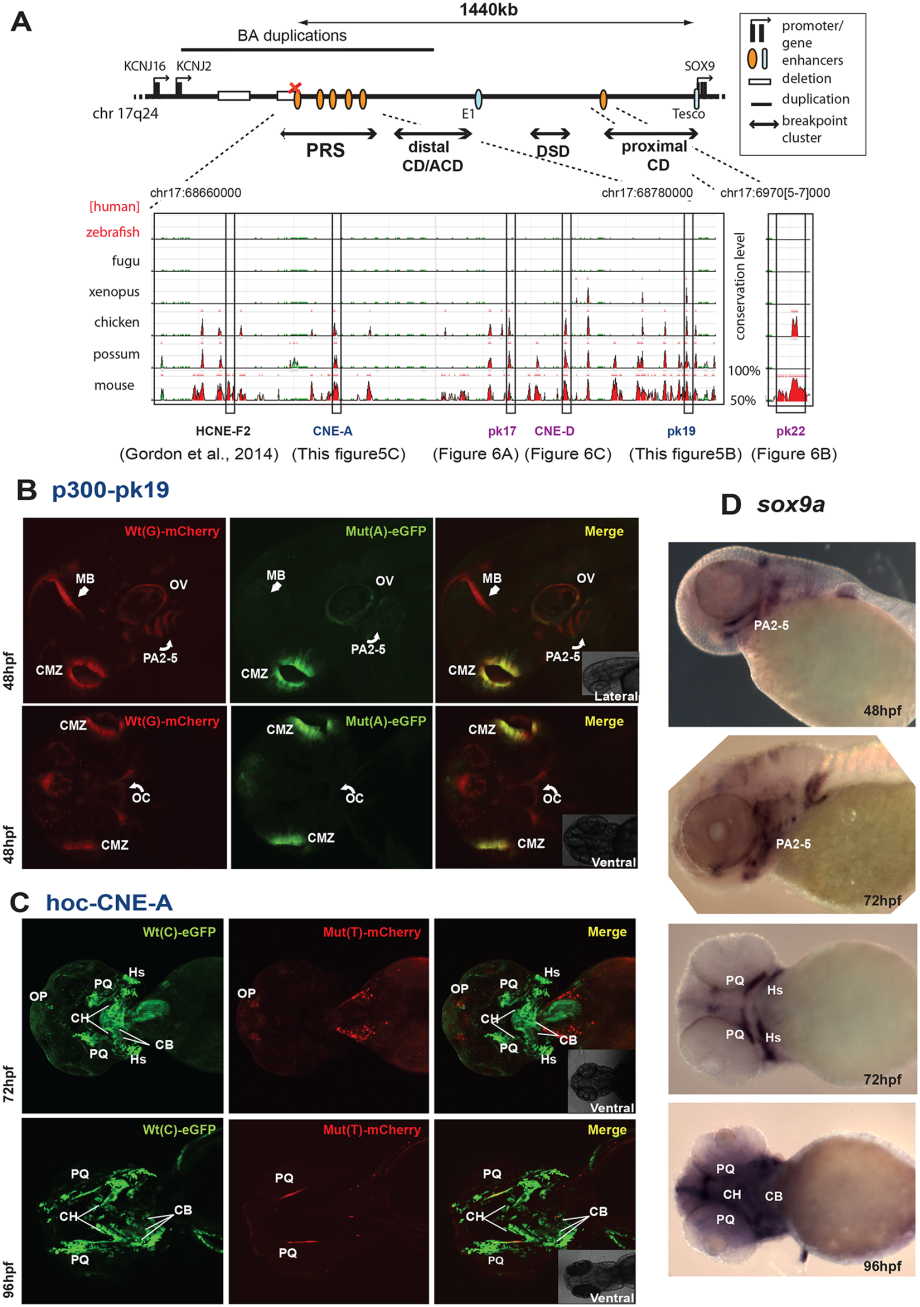
Characterisation of novel enhancers from the SOX9 genomic region with PRS-associated point mutations: spatial loss of expression. **(A)** Regulatory landscape of SOX9, depicting the location and sequence conservation across evolution for the novel SOX9 enhancers. **(B)** p300-PK19: Lateral and ventral views of F1 dual-fluorescence reporter transgenic embryos at 48 hpf. The Wt(G) allele drives expression in the region around the oral cavity (OC), pharyngeal arches (PA2-5), otic vesicle (OV), ciliary margin zone (CMZ) of the eye, and midbrain (MB). The Mut(A) allele has lost expression in the region around the oral cavity and pharyngeal arches (curved white arrow) and the midbrain (white arrow). **(C)** hoc-CNE-A: F1 dual-fluorescence reporter transgenic embryos at 72 hpf and 96 hpf. At 72 hpf the Wt(C) allele drives expression in the olfactory placodes (OP), and in the palatoquadrate (PQ), ceratohyal (CH), ceratobranchial (CB) (weak) and hyosymplectic (HS) cartilages. The Mut(T) allele has lost PQ, CH, CB and HS expression; OP expression is retained. At 96 hpf the Wt(C) allele drives expression in PQ, CH, CB. The Mut(T) allele has lost CH and CB expression, but retains reduced PQ expression. **(D)** RNA *in situ* hybridisation analysis of zebrafish *sox9a* at 48–96hpf showing overlap of reporter expression driven by the p300-Pk19 Wt(G) and hoc-CNE-A Wt(C) alleles with the endogenous *sox9a* expression pattern in the developing jaw. Wt: wild-type; Mut: mutant; hpf: hours post fertilization.

Having tested our assay system with a spectrum of previously described CREs implicated in disease, we embarked on the assessment of a set of novel predicted regulatory elements from the 1.5 Mb “gene desert” upstream of *SOX9*, in the interval between *SOX9* and *KCNJ2* (Fig 5A). Overlapping deletions associated with isolated Pierre Robin Sequence (PRS) were identified in the region 1.2–1.5 Mb upstream of SOX9, highlighting a sub-region particularly associated with PRS within the larger intergenic domain [7]. PRS is a craniofacial disorder characterized by micrognathia (mandibular hypoplasia), glossoptosis, and incomplete midline fusion of the palatal shelves typically leading to a U-shaped cleft palate. These features are thought to result as a causally linked sequence of developmental malformations arising from a primary deficiency in mandibular growth in early facial development. PRS often occurs as a component of Campomelic Dysplasia (CD;MIM114290), a syndrome affecting multiple tissues including cartilage, testes, notochord, neural crest, inner ear and the central nervous system. CD is caused by heterozygous loss-of-function coding mutations in the *SOX9* gene. In addition, a further group of CD cases result from disruption of regulatory control of *SOX9*. PRS can be considered an endo-phenotype of CD, caused by tissue-specific loss of full gene activity [17]. Detailed genetic analysis of a small set of PRS patients had identified a locus for isolated PRS at ~1.2–1.5 Mb upstream of *SOX9* [18], which has been refined using further patient studies [7]. Putative craniofacial regulatory elements potentially relevant to PRS etiology were identified in this region through a combination of sequence conservation (at least 70% identity over 300 bp between human, opossum and chick—the hoc elements), and by p300 ChIP profiling in mouse craniofacial tissue [7,19]. Sanger sequencing of eleven of the identified elements (Table 2) in a cohort of 69 individuals, mostly with isolated PRS, identified potentially causative private variants, not present in dbSNP137, in six of the elements studied. The genomic positions of the variant nucleotides in these elements, each showing considerable to high evolutionary conservation (as indicated by their GERP scores) (except for PRS135), and potential TF binding sites affected, are displayed in S2 Table. None of the identified variants could be shown to have arisen *de novo* in the patients; in two cases no parents were available for testing. The family pedigrees of the patients are shown in S4 Fig, with the sequenced individual arrowed and other identified carriers of the mutant allele noted. Incomplete penetrance and variable expressivity was observed in all the families studied.

**Table 2:** Novel PRS-associated elements tested for enhancer function.

SOX9 Reporter construct	Patient ID	CRE variant position chr 17 (hg19)	Distance from SOX9 promoter	Variant	Expression pattern in craniofacial tissues								Expression change in mutant.
		SOX9 ATG (70,117,533)	0		Oral Cavity (OC)	Meckel’s cartilage (MC)	Ethmoid plate (EP)	Ceratohyal (CH)	Ceratobranchials (CB)	Hyosymplectic (HS)	Pharyngeal arches (PA)	Palatoquadrate (PQ)	
hoc-CNE-A	PRS20	68,698,977	1,418,556	C>T Het	-	-	-	+	+	+	-	+	Lost in CH, CB and HS
p300-PK17	PRS26	68,735,530	1,382,003	G>A Het	+	+	-	+	-	-	-	+	Lost/reduced in OC, MC and PQ
hoc-CNE-D	PRS65	68,747,322	1,370,211	T>C Het	+	-	-	-	+	-	-	-	No change
p300-PK19	PRS100	68,772,750	1,344,783	G>A Het	+	-	-	-	-	-	+	-	Lost in OC and PA
p300-PK22	PRS135	69,706,044	411,489	A>C Het	+	-	-	-	-	-	-	-	No change
Combined Sox9a and Sox9b expression pattern in the developing craniofacial region of zebrafish embryos from 1–5dpf					+	+	+	+	+	+	+	+	

**Fig 6:**
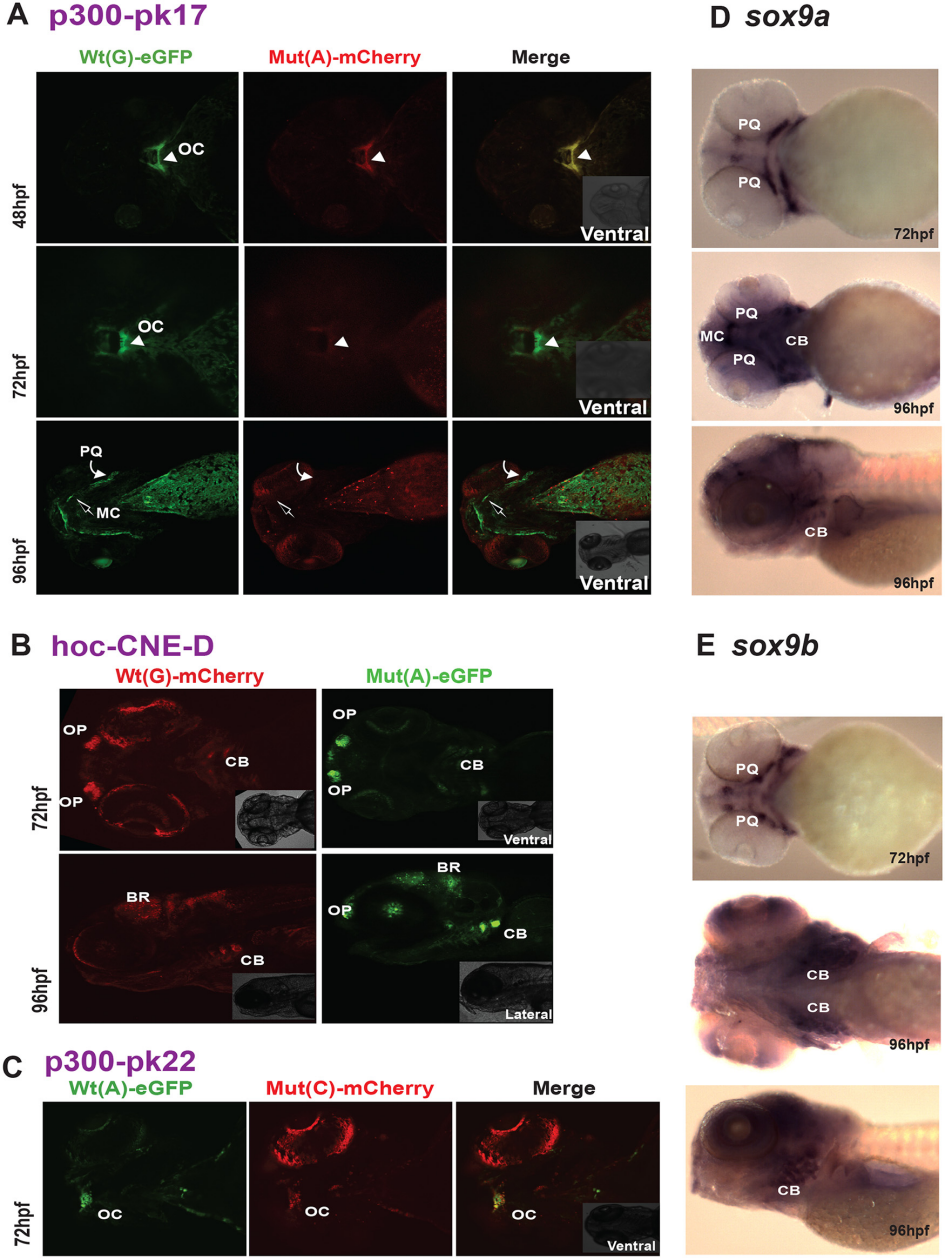
New enhancers from the SOX9 region with PRS-associated single nucleotide variants. **(A) p300-PK17**, spatiotemporally altered expression: F1 dual-fluorescence reporter transgenic embryos shown at 48, 72 and 96 hpf. At 48 and 72 hpf the Wt(G) allele drives expression in the region around the oral cavity (OC) (white arrowhead). At 96 hpf Wt(G) drives expression in the palatoquadrate (PQ) (curved white arrow) and Meckel’s cartilage (MC) (open arrow). The Mut(A) allele is able to drive OC expression at 48 hpf, but this is lost by 72 hpf and at 96 hpf no PQ and MC expression is observed. **(B) hoc-CNE-D**, tissue-specific CRE with unaltered expression: F1 dual-fluorescence transgenic embryos at 72 and 96 hpf. The Wt(T) and the Mut(C) alleles both drive expression in olfactory placode (OP), brain (BR), and ceratobranchials (CB). **(C) p300-PK22**, craniofacial CRE with unaltered expression: F1 dual-fluorescence transgenic embryos at 72 hpf. The Wt(A) and Mut(C) allele both drive expression in the region around the oral cavity (OC). **(D-E)** RNA *in situ* hybridisation analysis of zebrafish *sox9a* and *sox9b* at 72hpf and 96hpf, showing overlap of reporter gene expression driven by the p300-Pk17, hoc-CNE-D and p300-Pk22 elements with the endogenous *sox9a* and *sox9b* expression pattern in the developing jaw. Wt: wild-type; Mut: mutant; hpf: hours post fertilization.

We analysed five of the newly identified putative PRS associated CREs (Table 2) using our zebrafish assay, to establish potential enhancer function in plausible disease-related tissues that would be affected by the private SNVs in the PRS families (Figs 5 and 6). Remarkably, each of the tested CREs drives expression in specific craniofacial structures during development (Table 2 and S1 Table). Each element drives a distinct pattern, significantly overlapping with the overall expression pattern of *sox9* in zebrafish embryonic development (Figs 5D, 6D and 6E), and also with clear overlap between some of the elements.

We found that the PRS associated variants affect expression patterns in a variety of ways. Element p300-PK19 drives expression in the pharyngeal arches, midbrain, otic vesicle and ciliary margin zone of the eye. We observed the distinct tissue-specific loss of enhancer activity in pharyngeal arches 1–5 and in the midbrain of the Mut(A) allele of this element at 48 hpf (Fig 5B). However, expression was unaffected in the otic vesicle and the ciliary margin zone. Hoc-CNE-A drives extensive expression at major sites involved in the developing craniofacial apparatus of zebrafish embryos. At two different stages of development (72hpf and 96hpf), the Wt(C) allele shows expression in the palatoquadrate, ceratohyals, hyposymplectic, ceratobranchials and olfactory placodes. The Mut(T) allele however failed to drive expression in almost all the craniofacial tissues, partly retaining expression only in the palatoquadrate and olfactory placodes (Fig 5C).

We also observed temporal (and spatial) changes in tissue-specific CRE activity due to the presence of PRS-associated variants in another of the CREs (Fig 6A). P300-PK17 showed reporter expression in the first pharyngeal arch at 48 and 72 hpf. Expression driven by the mutant allele was present in the first pharyngeal arch at 48 hpf, but almost completely lost by 72 hpf. At 96 hpf reporter signal is seen in Meckel’s cartilage and the palatoquadrate in the wild-type but these sites are lost in the mutant (Fig 6A).

The final two CREs also revealed distinct craniofacial expression patterns in reporter transgenic embryos. However, for these elements we observed no differences in CRE activities between the wild-type and variant alleles (Fig 6B and 6C). Hoc-CNE-D was shown to drive expression in the ceratobranchials at two different stages of embryonic development (72 and 96 hpf) and showed additional sites of expression in the olfactory placodes and brain (Fig 6B). These sites of expression were also seen with the variant allele. The p300-PK22 construct showed expression at the base of the oral cavity with both the wild-type and the variant construct (Fig 6C). Thus our assay revealed craniofacial expression patterns for all five predicted CREs from the defined PRS region far upstream of *SOX9*, and altered spatiotemporal expression for three of these elements when carrying a sequence variant seen in patients with Pierre Robin Sequence.

## Discussion

The importance of intact gene regulation for correct development and homeostasis has become increasingly recognised. As a result, research groups interested in understanding the aetiology and causative mechanisms of human disorders or disease susceptibility are now routinely looking for possible mutations or variations in CREs in patient cohorts, especially in cases where convincing causative mutations could not be found in the coding regions of genes. However, the lack of efficient and physiologically relevant assays to functionally validate the consequence of these sequence changes and to distinguish disease-linked changes from background sequence variation is hampering the translation of knowledge about human genetic variation into an understanding of disease mechanisms. CRE activity is highly dependent on the availability of the right combination and stoichiometry of specific transcription factors. Consequently, high-throughput assays for assessing the potential effects of disease-associated SNPs in cultured cells are largely uninformative as they often lack the relevant and necessary biological context for tissue or cell-type specific CRE function. In future, cell lines reflecting more closely the cell phenotypes of developing tissues may become available through emerging stem cell differentiation protocols [20]. A few high-throughput *in vivo* assays, such as hydrodynamic tail-vein injection and retina explant assays [21–23] have also recently been developed but their application is restricted to CREs functional only in liver or retina respectively. Therefore there is a pressing need for robust, widely applicable, relatively high-throughput, cost effective and animal-number efficient *in vivo* assays that enable the unambiguous comparison of putative mutant and wild-type alleles of CREs for spatial and temporal differences in activity. Such screens are vital for differentiating between common, background sequence variation and functionally active, disease-associated mutations in CREs, and will enable rapid prioritization of candidates for functional follow-up studies.

The novel dual-colour zebrafish reporter transgenic assay pipeline that we have developed for investigating the functional consequences of disease- and disease-risk associated CRE mutations is designed to incorporate many of the above described merits, and thus will be a valuable tool for analysing effects of CRE mutations identified in patient cohorts and GWAS studies. While we cannot control for the number and position of transgene insertion sites in our assay, multiple independent transgenic lines bearing both the Wt and Mut version of the element driving distinct fluorescent reporters in the same animal can rapidly be constructed and tested, creating a clear consensus activity pattern and minimising the chance of bias in the analysis introduced by position effects due to site of integration of the transgenes. Other methods aimed towards targeted integration of transgenes in pre-defined docking sites in the zebrafish [24–27] or medaka [28] genomes are being developed, but the use of these techniques is more cumbersome and requires the maintenance of specialized fish stocks. Moreover, while targeted integration offers the advantage of avoiding bias from position effects, the use of dual-colour transgenesis is currently not established in these assays, complicating the possibility of simultaneous visualisation of CRE activity in the same animal, and precluding truly detailed assessment of differences in CRE activity. A comprehensive comparison of the merits and limitations of our assay with other available reporter transgenic assays for testing enhancer function is provided in S3 Table. We anticipate that it will become possible to combine advantages of the different methods in future assay designs.

Characterisation of detailed expression patterns driven by particular elements allows selection of the most likely candidate sequences by virtue of their expression in the relevant anatomical structures. In combination with information on the expression patterns of the genes located within the relevant genomic region this can also help to find the likely target gene of an enhancer. This can otherwise be problematic as target genes of enhancers are often not simply the nearest one in the genome, but can be found hundreds or even thousands of kb away in either direction, beyond intervening non-target genes, and may reside within the introns of other non-target genes [29, 30]. Most importantly our method enables the characterisation of wild-type and mutant variants of the same enhancer in the same individual animal. This is an important benefit for assessment of more subtle spatio-temporal differences as it allows a direct comparison without the need for extrapolation between separate individuals.

Disruption of cis-regulatory control of gene expression can occur via a number of distinct mechanisms [31]. These range from gross changes such as CRE deletion or translocations that separate the target gene from the influence of the CRE, to single nucleotide change in CREs that abrogate TF binding, or create novel binding sites. Although our method is suitable for a rapid screening of candidate regulatory sequences located in any small- to medium-sized region of interest that has been defined by deletion or translocation breakpoint mapping, the power of our analysis pipeline lies mostly in the characterisation of single nucleotide enhancer variants. We have therefore focussed on the latter mechanism, and our results indicate that it can be further subdivided based on the functional consequences of the basepair change. We observe at least four different types of effect on enhancer activity by patient mutant alleles. The first is the change in activity in the spatial dimension; this can manifest as complete loss of expression in a specific tissue (PAX6-SIMO, SOX9-hocCNEA), reduction in the extent of the expression domain (the SHH-SBE2 element), or an increase in expression domain (the SHH-ZRS). In the case of the SBE2 enhancer it had been shown that the reduction in expression domain size is caused by disruption of a SIX3 binding site in the element [9] and we replicated this effect by morpholino knock-down of Six3. This experiment demonstrates the modular nature of CREs: Six3 is not essential for all of the activity of SBE2 as expression in the caudal part of the hypothalamus remains, but Six3 is essential for the expression in the rostral hypothalamus.

The second mechanism we identify is a partial loss of activity in the temporal dimension, as exemplified by the p300-pk17 element in patient PRS026 which loses expression in the oral cavity only at later developmental stage. This indicates that the element functions normally when it first becomes activated, but fails to maintain its activity subsequently. The third example is the complete loss of functional activity of the element in our assay. This suggests the mutation disrupts the binding of an essential factor potentially affecting the effective formation of a TF complex, or enhanceosome [32] at the enhancer. The fourth type of outcome is an apparent lack of change, whereby the tissue-specific activity of the enhancer in the appropriate tissue is unaffected by the sequence variant. The causal connection to the disease phenotype in such cases is not obvious. The mutation could affect the precise level of transcriptional output from the element, which is not readily measured in the current fluorescent reporter assay, or it could disrupt some other function of the element not assayed here, for instance in organisation of chromatin conformation of the locus [33].

Even though not guaranteed to work for all enhancers, our results show that the dual reporter transgenic zebrafish assay is a suitable model system to test enhancer activity of many human disease-associated elements even when the element is not obviously conserved in zebrafish itself. Evolution is characterised by variable loss and gain of cis-regulatory sequences [34]. Teleost fish in particular appear to have been subject to an enhanced rate of evolutionary divergence due to the additional whole genome duplication (3R) that occurred at the base of the teleost lineage [35, 36], and consequently the zebrafish genome lacks many of the CNEs identifiable in other vertebrate species. However, it is likely that this increased divergence has occurred primarily at the level of the *cis*-regulatory sequences themselves, while the transcriptional machinery involved in the reading of the elements remains largely conserved due to selection against the wide-ranging effects of changes in the pleiotropic functions of transcription factors. Our results indicate that human regulatory elements implicated in disease can be tested in zebrafish with confidence.

It is remarkable that single nucleotide changes at such large genomic distance from their target gene can have such profound phenotypic effects. Most of the currently known examples of distal cis-elements involved in genetic disease act as enhancers for genes sensitive to haplo-insufficiency [31]. Our demonstration that single basepair mutations in multiple long-range cis-elements, with subtly different expression patterns, can lead to PRS highlights the sensitivity of craniofacial development to correct expression levels of SOX9. The same is true for the other genes, *SHH* and *IRF6*, for which enhancers were tested in this report.

Several of the enhancer variants described here are either inherited or even occur as rare SNPs in the wider population. Nevertheless we present strong evidence that these variants affect the function of the regulatory element in which they occur. Mutations in enhancers may not always lead to clear, fully penetrant phenotypes, but rather could contribute partly to a phenotype in specific individuals, depending on the presence of other modifier alleles. Incomplete penetrance may be observed for a number of reasons including variation in expressivity from the enhancer, or redundancy or buffering among multiple enhancers.

The mufti-faceted assay we have described here allows for the functional characterisation of disease-associated enhancer variants at the highest throughput that can currently be achieved using an *in vivo* animal model system. We anticipate that our strategy will facilitate the analysis of non-coding variants in genome-wide association study (GWAS) hits, and can also be used in studying the mechanistic basis for enhancer activity, the engineering of enhancers with desired properties, or to drive highly spatio-temporally specific expression of genetic tools.

## Materials and Methods

### Ethics statement

All zebrafish experiments were approved by the University of Edinburgh ethical committee and performed under UK Home Office license number PIL 60/12763; PPL 60/4418.

### Cloning of cis-regulatory elements for generating Tol2 constructs

CREs selected for analysis in transgenic reporter assays were cloned by PCR amplification of the fragment containing it plus flanking sequence from genomic DNA, using Phusion high fidelity polymerase (NEB). attB4 and attB1r sequences were included in the PCR primers for use with the Gateway recombination cloning system (Invitrogen). The amplified fragment was first cloned into the Gateway pP4P1r entry vector and sequenced using M13 forward and reverse primers for verification. In cases where a point mutation was engineered in the wildtype CRE, a site-directed mutagenesis kit (QuikChange II XL Site-Directed Mutagenesis Kit, Agilent) was used on the pP4P1r vector containing the wildtype CRE sequence. Test elements in the pP4P1r vector were combined with a pDONR221 construct containing either a gata2 promoter-eGFP-polyA or a gata2 promoter mCherry- polyA cassette, and recombined into a destination vector with a Gateway R4-R2 cassette flanked by Tol2 recombination sites [8, 37]. The primer sequences used for amplification of the published CREs described in the manuscript are listed in S4 Table and images from independent lines for some of the constructs are presented in S1 Fig.

### Patient sequencing

Purification of genomic DNA from blood lymphocytes and Sanger sequencing of non-coding elements upstream of SOX9 was performed according to standard protocols, using the primers listed in S4 Table.

### CRE sequence conservation

Sequence conservation of disease associated CREs was assessed by multiple sequence alignment and visualised using the VISTA or ECR Browser with the human hg19 sequence as base genome [38, 39].

### Generation of zebrafish transgenic lines

Zebrafish were maintained in a recirculating water system according to standard protocol [40]. Embryos were obtained by breeding adult fish of standard stains (AB and WIK) and raised at 28.5°C as described [40]. Embryos were staged by hours post fertilization (hpf) as described [41]. Reporter plasmids were isolated using Qiagen miniprep columns and were given extra purification via a Qiagen PCR purification column (Qiagen), and diluted to 50 ng/microliter with DNAse/RNAse free water. tol2 transposase RNA was synthesized from a NotI-linearized pCS2-TP plasmid [42] using the SP6 mMessage mMachine kit (Ambion), and similarly diluted to 50 ng/microliter. Equal volumes of the reporter construct(s) and the transposase RNA were mixed immediately prior to injections. 1–2 nl of the solution was micro-injected per embryo of up to 200 embryos at the 1- to 2-cell stage. Embryos were screened for mosaic fluorescence at 1–5 days post-fertilization i.e. 24–120 hpf (hours post fertilization) and raised to adulthood. Germline transmission was identified by mating of sexually mature adults to wild-type fish and examining their progeny for fluorescence. F1 embryos from 3–5 F0 lines showing the best representative expression pattern for each construct were selected for confocal imaging. A few positive embryos were also raised to adulthood and F1 lines were maintained by outcrossing. A summary of the number of independent lines analyzed for each construct and their expression sites is included in S1 Table.

### Imaging of zebrafish reporter transgenic embryos

Embryos for imaging were treated with 0.003% PTU (1-phenyl2-thio-urea) from 24 hpf to prevent pigmentation. Embryos selected for imaging were anaesthetized with tricaine and mounted in 1% low-melting agarose. Images were taken on a Nikon A1R confocal microscope and processed using A1R analysis software.

### Morpholino knock-down of Six3

A zebrafish Six3 antisense morpholino oligonucleotide (Six3AMO) was obtained from Gene Tools, LLC, with the following sequence: 5’ GCTCTAAAGGAGACCTGAAAACCAT 3’. This morpholino has sequence complementary to the highly conserved sequences around the translation initiation codon of both six3a and six3b, and hence inhibits the function of both zebrafish six3 genes [11]. As control we used the Gene ToolsLLC standard negative control morpholino: 5’ CCTCTTACCTCAGTTACAATTTATA 3’. The morpholinos were injected into 1 to 2-cell stage of at least 100 embryos to deliver an approximate amount of 2.5 ng per embryo.

### mRNA *in situ* hybridization

RNA *in situ* hybridization on fish embryos was performed as previously described [43]. The sequences of primers used for synthesis of hybridization probes are listed in S5 Table.

### Western blotting

Nuclear extract was prepared from ~100 morpholino injected embryos at 48hpf using NE-PER Nuclear and Cytoplasmic Extraction Reagents (Thermo Scientific, catalogue number 78833). The extracts were boiled with 30 μL of loading buffer (12.5mM Tris at pH 6.8, 2% SDS, 20% glycerol, 0.002% bromphenol blue, 10% 2-mercaptoethanol) for 5min and were resolved by 10% SDS-PAGE, transferred to nitrocellulose, incubated with antibody (anti-Six3 antibody ab139317), and detected by chemiluminescence (Thermo scientific SuperSignal West Femto Maximum Sensitivity Substrate). The membrane was then re-probed with anti-α-tubulin antibody (ab44928) as loading control.

## Supporting Information

S1 Fig Wild type and variant CRE driven transgene expression patterns in F1 embryos obtained from multiple independent stable transgenic lines. Dual reporter fluorescence in transgenic F1 embryos from multiple additional independent lines for the SHH-SBE2 (A), SHH-ZRS (B), PAX6-SIMO (C), p300-Pk19 (D) and p300-Pk17 (E) elements. These lines are independent from the ones shown in the main text. The expression sites that are consistent between the multiple transgenic lines for each of the CREs are marked. RH: rostral hypothalamus; CH: caudal hypothalamus; PF: pectoral fin; L: lens; CMZ: ciliary margin zone; OV: otic vesicle; PA2-5: pharyngeal arch 2–5; OC: oral cavity; PQ: palatoquadrate; MC: Meckel’s cartilage; Wt: wild-type; Mut: mutant; hpf: hours post fertilization.
(TIF)

S2 Fig Western blot to confirm morpholino mediated Six3 knockdown. The efficiency of Six3 depletion in zebrafish embryos injected with Six3 AMO morpholinos is demonstrated by Western blotting of protein extract from pooled morpholino-injected embryos with anti-Six3 antibody. The absence of Six3 in the extract from Six3 AMO morpholino-injected embryos is highlighted by a black arrow at 32 KDa, while Six3 is present in fish injected with a control morpholino. After stripping, the blot was incubated with anti-α-tubulin antibody for loading control (50 kDa, grey arrow).
(TIF)

S3 Fig SHH-ZRS in multiple founders. SHH-ZRS enhancer-driven reporter expression is shown at 72 hpf **(A)** The Wt(G) allele is shown driving eGFP expression in a restricted region of the developing pectoral fin (PF) in two independent founders (1 and 2). **(B)** The Wt(G) allele is shown driving mCherry expression in a restricted region of the developing pectoral fin (PF) in four independent founders (1–4). Consistent reporter gene expression was detected only in the pectoral fin in all independent founders shown.
(TIF)

S4 Fig Pedigrees for Pierre Robin sequence (PRS) patients harbouring candidate pathogenic variants in SOX9 non-coding elements. Genotypes are indicated for those individuals available for sequencing: + wildtype allele,—mutant allele. Probands are indicated with an arrow.
(TIF)

S1 Table Wild type and variant CRE driven transgene expression sites in F1 embryos obtained from multiple independent stable transgenic F0 lines. (DOCX)

S2 Table Candidate pathogenic variants identified in conserved non-coding elements within the PRS-region upstream of SOX9 in PRS patients. (DOCX)

S3 Table Summary of pros and cons of the dual-colour zebrafish reporter transgenic CRE assay versus PhiC31-integrase mediated targeted integration assays. (DOCX)

S4 Table Primers used to prepare the CRE constructs. (DOCX)

S5 Table Primers used to prepare the probes for RNA *in situ* hybridisation. (DOCX)
