# Correction: Functional Assessment of Disease-Associated Regulatory Variants *In Vivo* Using a Versatile Dual Colour Transgenesis Strategy in Zebrafish

**DOI:** 10.1371/journal.pgen.1005572

**Published:** 2015-10-15

**Authors:** The PLOS Genetics Staff

## Notice of Republication

This article was republished on June 24, 2015, to correct an error in the XML that was causing Veronica van Heyningen’s name to appear incorrectly in PubMed. The publisher apologizes for this error. The pdf of the article has not changed.
