# Supplementary figures and images for: Functional Assessment of Disease-Associated Regulatory Variants In Vivo Using a Versatile Dual Colour Transgenesis Strategy in Zebrafish
Source: PLoS Genet. 2015 Jun 1;11(6):e1005193. doi: 10.1371/journal.pgen.1005193 (PMC4452300; doi:10.1371/journal.pgen.1005193)

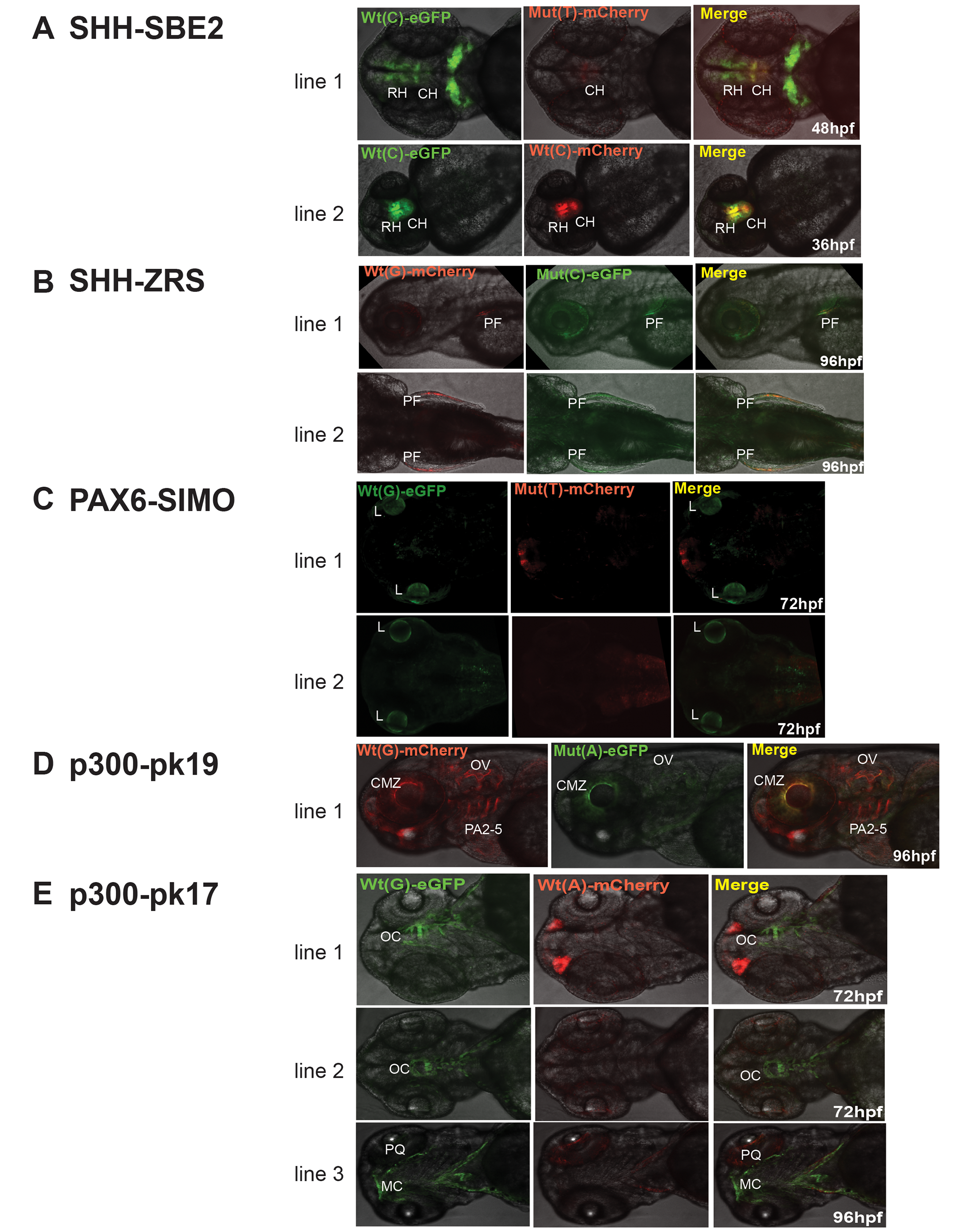

Supplement: S1 Fig — Dual reporter fluorescence in transgenic F1 embryos from multiple additional independent lines for the SHH-SBE2 (A), SHH-ZRS (B), PAX6-SIMO (C), p300-Pk19 (D) and p300-Pk17 (E) elements. These lines are independent from the ones shown in the main text. The expression sites that are consistent between the multiple transgenic lines for each of the CREs are marked. RH: rostral hypothalamus; CH: caudal hypothalamus; PF: pectoral fin; L: lens; CMZ: ciliary margin zone; OV: otic vesicle; PA2-5: pharyngeal arch 2–5; OC: oral cavity; PQ: palatoquadrate; MC: Meckel’s cartilage; Wt: wild-type; Mut: mutant; hpf: hours post fertilization. (TIF) [file pgen.1005193.s001.tif]

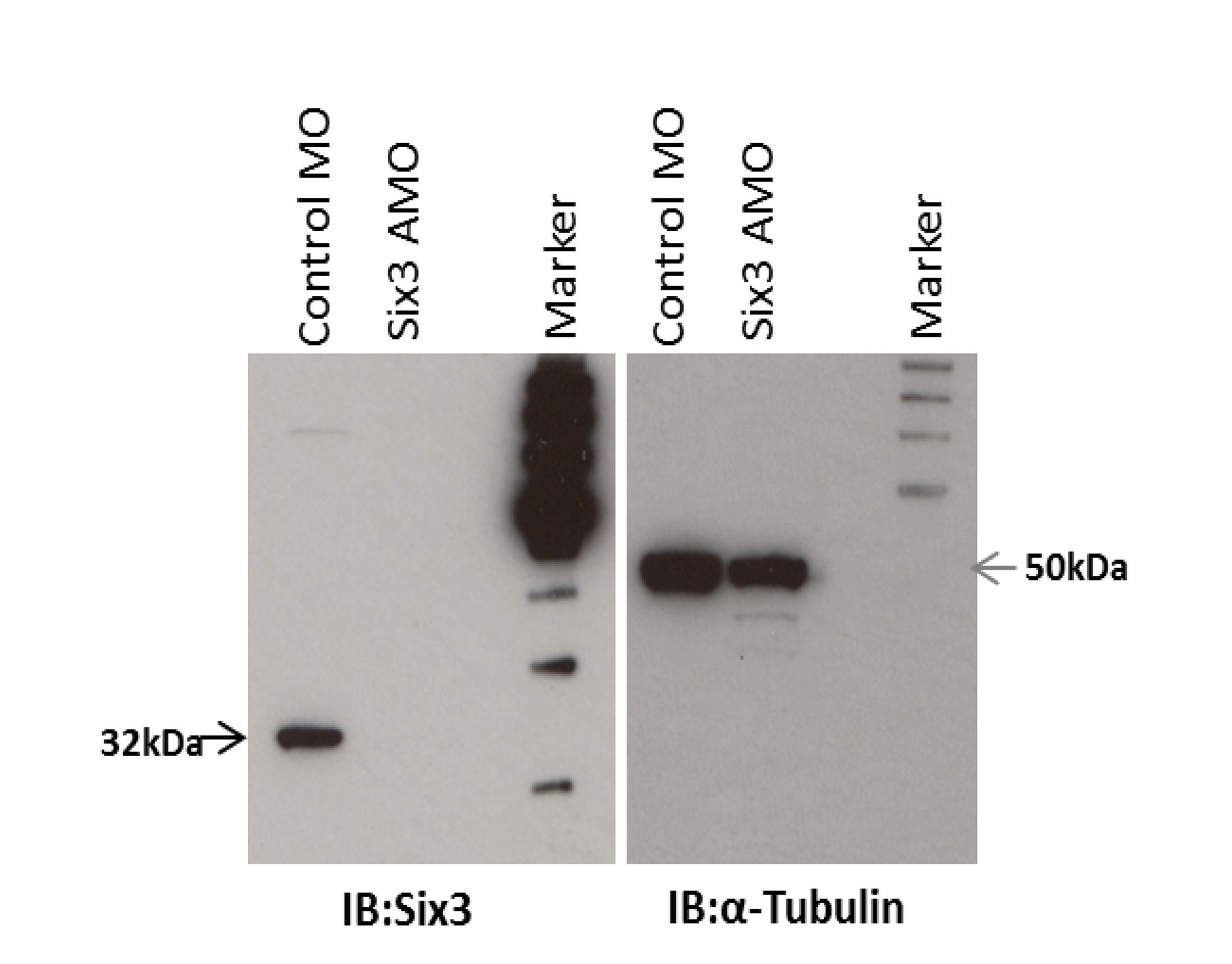

Supplement: S2 Fig — The efficiency of Six3 depletion in zebrafish embryos injected with Six3 AMO morpholinos is demonstrated by Western blotting of protein extract from pooled morpholino-injected embryos with anti-Six3 antibody. The absence of Six3 in the extract from Six3 AMO morpholino-injected embryos is highlighted by a black arrow at 32 KDa, while Six3 is present in fish injected with a control morpholino. After stripping, the blot was incubated with anti-α-tubulin antibody for loading control (50 kDa, grey arrow). (TIF) [file pgen.1005193.s002.tif]

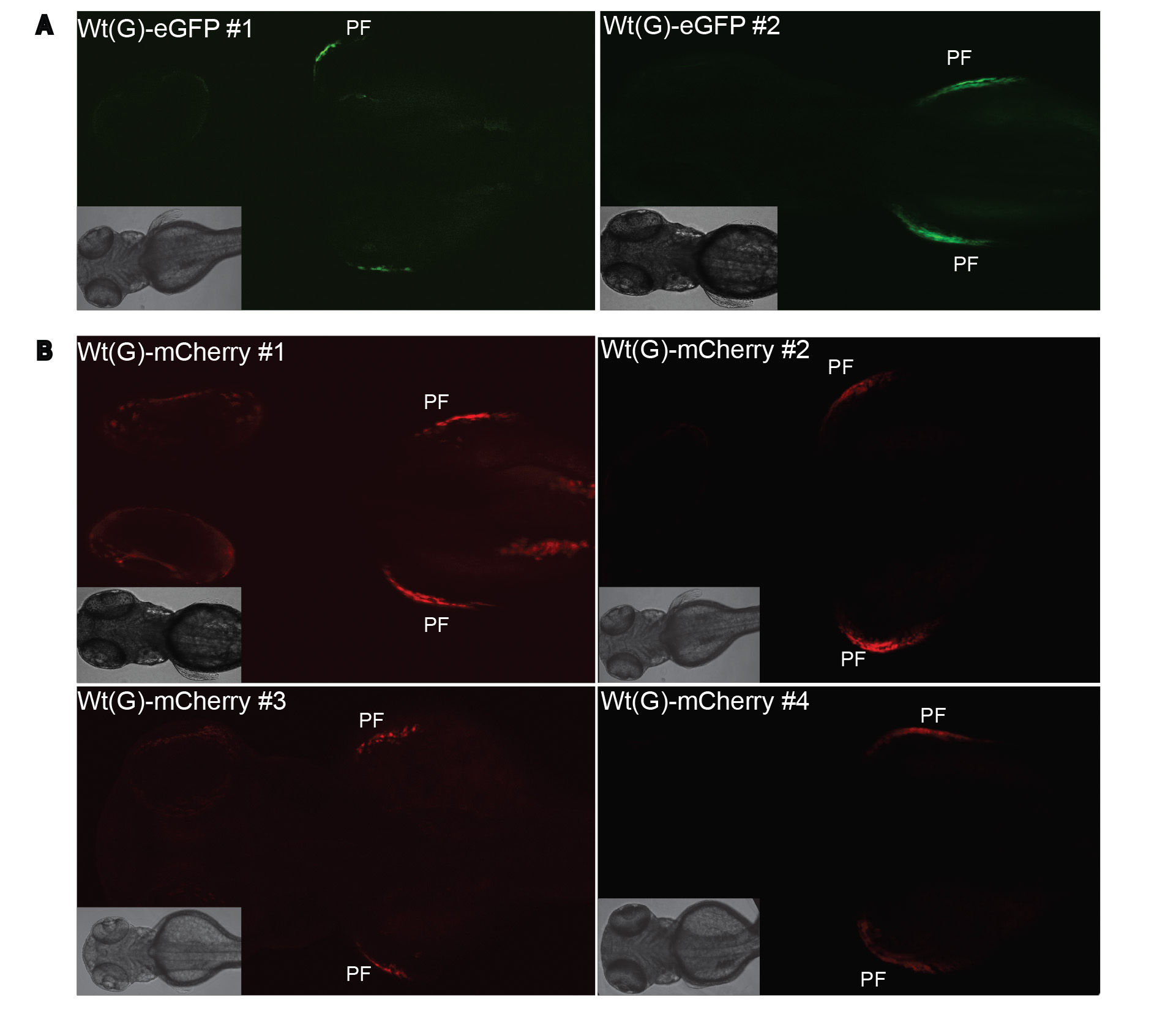

Supplement: S3 Fig — SHH-ZRS enhancer-driven reporter expression is shown at 72 hpf (A) The Wt(G) allele is shown driving eGFP expression in a restricted region of the developing pectoral fin (PF) in two independent founders (1 and 2). (B) The Wt(G) allele is shown driving mCherry expression in a restricted region of the developing pectoral fin (PF) in four independent founders (1–4). Consistent reporter gene expression was detected only in the pectoral fin in all independent founders shown. (TIF) [file pgen.1005193.s003.tif]

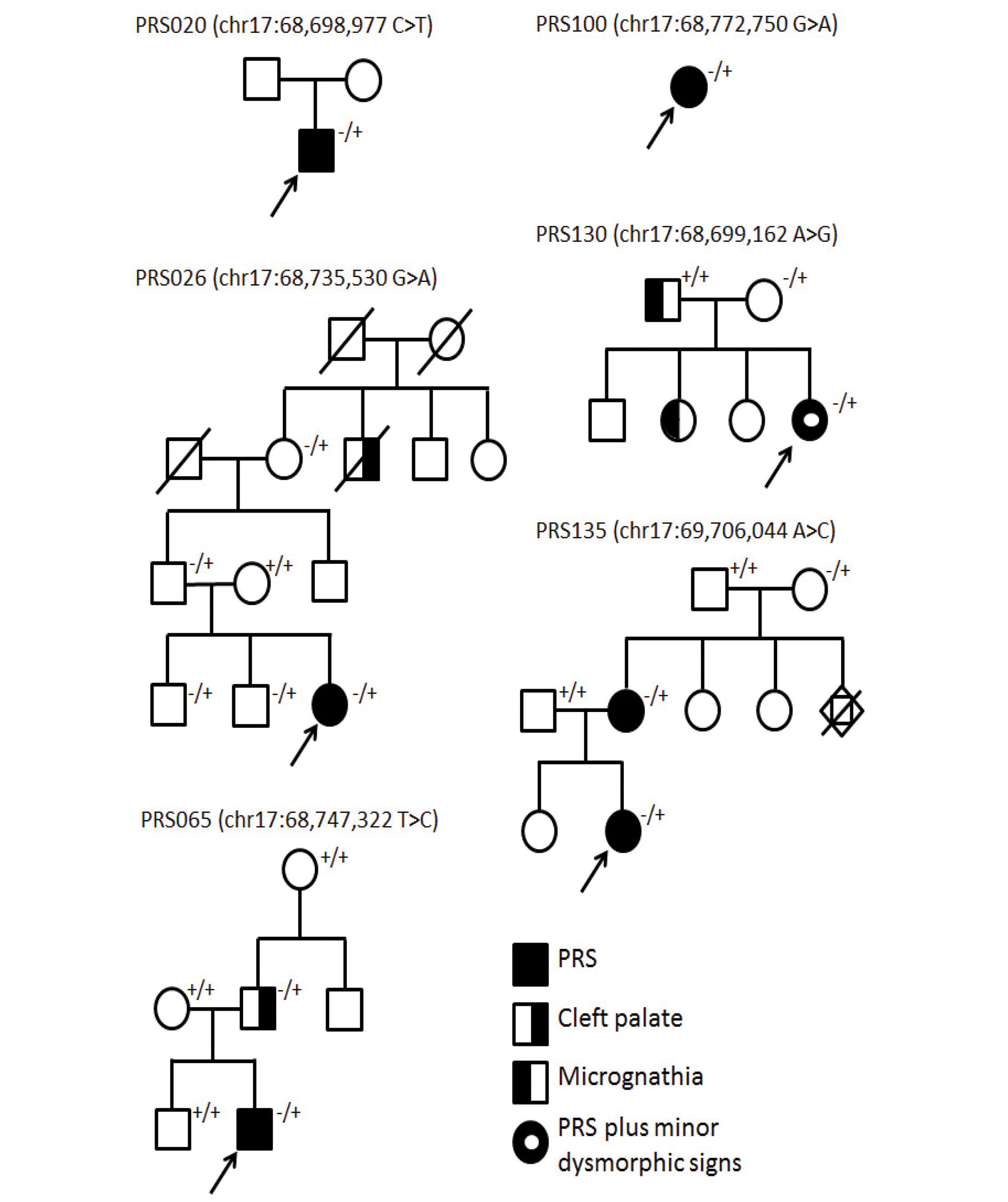

Supplement: S4 Fig — Genotypes are indicated for those individuals available for sequencing: + wildtype allele,—mutant allele. Probands are indicated with an arrow. (TIF) [file pgen.1005193.s004.tif]
