# Supplementary material for: Functional Assessment of Disease-Associated Regulatory Variants In Vivo Using a Versatile Dual Colour Transgenesis Strategy in Zebrafish
Source: PLoS Genet. 2015 Jun 1;11(6):e1005193. doi: 10.1371/journal.pgen.1005193 (PMC4452300; doi:10.1371/journal.pgen.1005193)
Supplement: S1 Table — (DOCX) [file pgen.1005193.s005.docx]

**S1 Table:** **Wild type and variant CRE driven transgene expression sites in F1 embryos obtained from multiple independent stable transgenic F0 lines.**

| Transgene * | Reporter used in transgenic assay ^#^ | Total number of stable transgenic lines analysed | Sites of reporter expression  driven by the element | Tissue-specific activity of the CRE observed in 100% of transgenic lines analysed |
| --- | --- | --- | --- | --- |
| Shh-SBE2-WT(C) | eGFP | 4 | **Rostral hypothalamus**  **(4/4; 100%)**  **Caudal hypothalamus**  **(4/4; 100%)**  Otic vesicle (1/4; 25%)  Heart (1/4; 25%)  Jaw (1/4;25%) | Rostral and caudal hypothalamus |
| Shh-SBE2-Mut(T) | mCherry | 4 | Rostral hypothalamus (0/4; 0%)  **Caudal hypothalamus**  **(4/4; 100%)**  Otic vesicle (1/4; 25%)  Olfactory placode (1/4; 25%) | Caudal hypothalamus |
| Shh-SBE2-WT(C) | mCherry | 3 | **Rostral hypothalamus**  **(3/3; 100%)**  **Caudal hypothalamus**  **(3/3; 100%)**  Otic vesicle (1/3; 33%) | Rostral and caudal hypothalamus |
| Shh-SBE2-Mut(T) | eGFP | 4 | Rostral hypothalamus (0/4; 0%)  **Caudal hypothalamus**  **(4/4; 100%)**  Heart (1/4; 25%) | Caudal hypothalamus |
| Shh-ZRS-WT(G) | eGFP | 3 | **Pectoral fin (3/3 ; 100%)**  Yolk sac (1/3 ; 33%)  Lens(1/3 ; 33%)  Pelvic fin (1/3; 33%) | Pectoral fin |
| Shh-ZRS-WT(G) | mCherry | 4 | **Pectoral fin (4/4; 100%)**  Olfactory placode (1/4; 25%) | Pectoral fin |
| Shh-ZRS-Mut(A) | eGFP | 4 | **Pectoral fin (4/4; 100%)**  Otic vesicle (1/4; 25%)  Retina (1/4; 25%)  Heart (1/4; 25%) | Pectoral fin |
| PAX6-SIMO(G) | eGFP | 3 | **Lens (3/3; 100%)**  Otic vesicle (1/3; 33%)  Pectoral fin (1/3; 33%)  Forebrain (1/4; 25%)  Hindbrain (1/4;25%) | Lens |
| PAX6-SIMO(G) | mCherry | 4 | **Lens (4/4; 100%)**  Otic vesicle (1/4; 25%)  Heart (1/4; 25%) | Lens |
| PAX6-SIMO(T) | eGFP | 4 | Lens (0/4; 0%)  Forebrain (1/4; 25%) | - |
| PAX6-SIMO(T) | mCherry | 3 | Lens (0/4; 0%)  Forebrain (1/4; 25%)  Retina (1/4; 25%)  Forebrain (1/4;25%)  Neural tube (1/4; 25%) | - |
| IRF6-MCS9.7-WT(G) | mCherry | 4 | **Ethmoid plate (4/4; 100%)**  **First pharyngeal arch**  **(4/4; 100%)**  Pectoral fins (1/4; 25%)  Retina (1/4; 25%) | Ethmoid plate and first pharyngeal arch |
| IRF6-MCS9.7-Mut(A) | eGFP | 4 | Ethmoid plate (0/4; 0%)  **First pharyngeal arch**  **(4/4; 100%)**  Yolk sac (1/4; 25%) | First pharyngeal arch |
| p300-PK19-WT(G) | mCherry | 4 | **Ciliary margin zone (4/4; 100%)**  **Midbrain (4/4; 100%)**  **Pharyngeal arches (4/4; 100%)**  **Otic vesicle (4/4; 100%)**  Yolk sac (1/4; 25%)  Heart (1/4;25%) | Ciliary margin zone, midbrain, pharyngeal arches and otic vesicle |
| p300-PK19-Mut(A) | eGFP | 4 | **Ciliary margin zone (4/4; 100%)**  Midbrain (0/4; 0%)  Pharyngeal arches (0/4; 0%)  **Otic vesicle (4/4; 100%)**  Diencephalon (1/4; 25%) | Ciliary margin zone and otic vesicle |
| hoc-CNE-A-WT(C) | eGFP | 4 | **Palatoquadrate (4/4; 100%)**  **Ceratohyal (4/4; 100%)**  **Hyosymplectic (4/4; 100%)**  **Ceratobrachials (4/4; 100%)**  Olfactory placode (2/4; 50%)  Heart (1/4; 25%) | Palatoquadrate, ceratohyal, hyosymplectic and ceratobrachials |
| hoc-CNE-A-Mut(T) | mCherry | 4 | Palatoquadrate (0/4; 0%)  Ceratohyal (0/4; 0%)  Hyosymplectic (0/4; 0%)  Ceratobrachials (0/4; 0%)  Olfactory placode (3/4; 75%)  Yolk sac (1/4; 25%) | - |
| p300-PK17-WT(G) | eGFP | 4 | **First pharyngeal arch**  **(4/4; 100%)**  **Palatoquadrate (4/4; 100%)**  **Meckel’s cartilage (4/4; 100%)**  Lens (1/4; 25%)  Forebrain (1/4; 25%)  Yolk sac (1/4; 25%) | First pharyngeal arch, palatoquadrate and meckel’s cartilage |
| p300-PK17-Mut(A) | mCherry | 4 | **First pharyngeal arch**  **(4/4; 100%)**  Palatoquadrate (0/4; 0%)  Meckel’s cartilage (0/4; 0%)  Lens (1/4; 25%)  Yolk sac (2/4; 50%)  Olfactory placode (1/4; 25%)  Retina (1/4;25%) | First pharyngeal arch |
| hoc-CNE-D-WT(T) | mCherry | 4 | **Forebrain (4/4; 100%)**  **Olfactory placode (4/4; 100%)**  **First pharyngeal arch**  **(4/4; 100%)**  **Ceratobrachials (4/4; 100%)**  Lens (1/4; 25%) | Forebrain, olfactory placode, first pharyngeal arch and ceratobrachials |
| hoc-CNE-D-Mut(C) | eGFP | 4 | **Forebrain (4/4; 100%)**  **Olfactory placode (4/4; 100%)**  **First pharyngeal arch**  **(4/4; 100%)**  **Ceratobrachials (4/4; 100%)**  Lens (2/4; 50%)  Retina (1/4; 25%) | Forebrain, olfactory placode, first pharyngeal arch and ceratobrachials |
| p300-PK22-WT(A) | eGFP | 4 | **First pharyngeal arch**  **(4/4; 100%)**  Lens (1/4; 25%) | First pharyngeal arch |
| p300-PK22-Mut(C) | mCherry | 3 | **First pharyngeal arch**  **(3/3; 100%)** | First pharyngeal arch |

* Species of origin: *Homo sapiens*

# Model organism for reporter transgenic assay: *Danio rerio*

|  |  |  |  |  |
| --- | --- | --- | --- | --- |
|  |  |  |  |  |
|  |  |  |  |  |
|  |  |  |  |  |
|  |  |  |  |  |
|  |  |  |  |  |
|  |  |  |  |  |
|  |  |  |  |  |
|  |  |  |  |  |
|  |  |  |  |  |
