# Supplementary material for: Functional Assessment of Disease-Associated Regulatory Variants In Vivo Using a Versatile Dual Colour Transgenesis Strategy in Zebrafish
Source: PLoS Genet. 2015 Jun 1;11(6):e1005193. doi: 10.1371/journal.pgen.1005193 (PMC4452300; doi:10.1371/journal.pgen.1005193)
Supplement: S2 Table — (DOCX) [file pgen.1005193.s006.docx]

**S2 Table:** **Candidate pathogenic variants identified in conserved non-coding elements within the PRS-region upstream of SOX9 in PRS patients.**

| **Potential**  **CRE** | **Patient** | **Position**  **(Hg19)** | **Description** | **GERP**  **score (#)** | **Falls within HMR TFBS (&)** |
| --- | --- | --- | --- | --- | --- |
| hoc-CNE-A | PRS20 | 68698977 | C>T Het | 4,75 | V$OCT1_01 |
| p300-PK17 | PRS26 | 68735530 | G>A Het | 4,47 | no |
| hoc-CNE-D | PRS65 | 68747322 | T>C Het | 2,68 | V$NFAT_Q6 |
| p300-PK19 | PRS100 | 68772750 | G>A Het | 3,53 | V$CDP_01 |
| hoc-CNE-A | PRS130 | 68699162 | A>G Het | 5,58 | V$OCT1_07, V$EVI1_03, V$LMO2COM_02, V$GATA6_01, V$GATA1_05 |
| p300-PK22 | PRS135 | 69706044 | A>C Het | 0,00149 | no |
