# Supplementary material for: Functional Assessment of Disease-Associated Regulatory Variants In Vivo Using a Versatile Dual Colour Transgenesis Strategy in Zebrafish
Source: PLoS Genet. 2015 Jun 1;11(6):e1005193. doi: 10.1371/journal.pgen.1005193 (PMC4452300; doi:10.1371/journal.pgen.1005193)
Supplement: S3 Table — (DOCX) [file pgen.1005193.s007.docx]

**S3 Table: Summary of pros and cons of the dual-colour zebrafish reporter transgenic CRE assay vs PhiC31-integrase mediated targeted integration assays.**

| **Dual colour Tol2-mediated transgenesis** | |
| --- | --- |
| **Advantages** | Activities of Wt and Mut CREs compared directly in the F1 embryos obtained from founder (F0) fish (Analysis in F0 fish possible, but hampered by cellular mosaicism)  Co-injection and stable integration of both CRE-reporter constructs in the same founder significantly reduces the effects of mosaicism in F1s and brings both alleles in the same F1 embryo at high frequency  Rapid generation of CRE-reporter constructs using Gateway cloning strategy, massively simplifying and speeding up the time required to clone the Wt and Mut elements  Germline transmission rate of 20%  Live imaging for detection of temporal differences between Wt vs Mutant CRE alleles over the entire time course of embryonic development possible in F1 embryos  Analysis of altered transcription factor binding potential of mutant CRE allele possible by simple morpholino-mediated knockdown in F1 embryos |
| **Limitations** | Detection of quantitative differences between Wt and Mut-CRE alleles not possible with current method due to variability in number and site of integration of the two constructs  Integration of the transgenes occurs at random sites in the genome. |
| **PhiC31-integrase mediated transgenesis** | |
| **Advantages** | Detection of quantitative differences between Wt and Mut-CRE alleles possible due to single copy site-specific integration of each allele  Stable lines with defined integration sites for the transgenes can be obtained  Analysis of altered transcription factor binding potential of mutant CRE allele possible by simple morpholino-mediated knockdown in F1 embryos |
| **Limitations** | Two different recipient fish lines required to enable the assessment of Wt and Mutant CRE- reporter constructs  Simultaneous comparison of the Wt and Mut CRE activities requires breeding of the individual founder (F0) fish. Obtaining both alleles in the same F1 embryo would be significantly impeded by the mosaicism in the individual F0’s and the dual-colour analysis would only be successfully performed in the F2 generation  Standard cloning methods utilized for generation of CRE-reporter constructs impeding the speed of the assay  Germline transmission rate of 10% (21)  Live imaging for detection of temporal differences between Wt vs Mutant CRE alleles over the entire time course of embryonic development only possible in F2 embryos. |
