# Supplementary material for: Functional Assessment of Disease-Associated Regulatory Variants In Vivo Using a Versatile Dual Colour Transgenesis Strategy in Zebrafish
Source: PLoS Genet. 2015 Jun 1;11(6):e1005193. doi: 10.1371/journal.pgen.1005193 (PMC4452300; doi:10.1371/journal.pgen.1005193)
Supplement: S4 Table — (DOCX) [file pgen.1005193.s008.docx]

**S4 Table:** **Primers used to prepare the CRE constructs.**

| **CRE** | **hg19 coordinates** | **Length (bp)** | **Forward primer**  **(5’-attB4-sequence – 3’)** | **Reverse primer**  **(5’-attB1r-sequence – 3’)** |
| --- | --- | --- | --- | --- |
| Shh-SBE2 | chr7:156061034-156062258 | 1225 | GCCTAGCGTTTCCAACATGCAGCC | TACGGCTCTAACAGTAAAGCACTC |
| Shh-ZRS | chr7:156583546- 156584949 | 1400 | ACGCCCAGATTTGATGTTTC | GCTCTTAGGGCTCAGGGAAC |
| PAX6-SIMO | chr11:31685344-31686179 | 830 | GGCGCGCCTTTCTCAGGAAGAAATCAGGT | CTATAGGATGCTGTAGTTGG |
| IRF6-MCS9.7 | chr1:209989217-209989470 | 254 | GATTGGAGCTTTGGAATGTTAATC | ACAGAATAGTAACTGGGAGGTTGC |
| SOX9 hoc-CNE-A | chr17:68698215-68699574 | 1360 | CAGTGCCCTCAGTCAGGTCT | TTACCCTTACCCTCCAGCTGT |
| SOX9-p300-PK17 | chr17:68735149-68735915 | 766 | CATAATGACAGTGACCAGTG | GCCCTCCTGAGGAAGAGTG |
| SOX9-hoc-CNE-D | chr17:68746903-68747865 | 963 | TGGGCTCATTTCTTCAGG | GCAATGACACAAGCCGTG |
| SOX9- p300-PK19 | chr17:68772528-68773259 | 732 | CTTCATCCTATTTTCACTTCC | ACCCACAAAATCACCTGGAG |
| SOX9- p300-PK22 | chr17:69705773-69706609 | 837 | CCGTTGTGTGCTGAATGG | GGGCTACTCACTGCTTCTCCA |
