# Supplementary material for: Functional Assessment of Disease-Associated Regulatory Variants In Vivo Using a Versatile Dual Colour Transgenesis Strategy in Zebrafish
Source: PLoS Genet. 2015 Jun 1;11(6):e1005193. doi: 10.1371/journal.pgen.1005193 (PMC4452300; doi:10.1371/journal.pgen.1005193)
Supplement: S5 Table — (DOCX) [file pgen.1005193.s009.docx]

**S5 Table:** **Primers used to prepare the probes for RNA *in situ* hybridisation.**

| **Gene** | **Forward primer**  **(5’-SP6 promoter-sequence – 3’)** | **Reverse primer**  **(5’-T7 promoter-sequence – 3’)** |
| --- | --- | --- |
| *shha* | AAGCTGACACCTCTCGCCTA | GAGCAATGAATGTGGGCTTT |
| *shhb* | GACGGTGACACTTGGTGATG | ATGTGCCCATTTGTGGTTCT |
| *pax6a* | CCCAATACTGGCCCAGACTA | AAGTGGCACTATCCCC |
| *irf6* | GCTGGTCTGGCTGGATAGAG | GACGGCTGAAGAGGAGACAC |
| *sox9a* | CACGTCAAGAGACCGATGAA | TCTCGTTTCAGATCCGCTTT |
| *sox9b* | GAGCGAGAGAGAGCGAGAGA | ATTCCCTCAGAGCACGCTAA |
